# Neuroprotective Effects of Intermittent Theta Burst Stimulation in Parkinson’s Disease (NET-PD): A Study Protocol for a Delayed-Start Randomized Double-Blind Sham-Controlled Trial

**DOI:** 10.3390/jcm11174972

**Published:** 2022-08-24

**Authors:** Puyu Li, Ningdi Luo, Sainan Sun, Yuanyuan Li, Dingding Shen, Xue Zhu, Liche Zhou, Haiyan Zhou, Jun Liu

**Affiliations:** 1 Department of Neurology, Institute of Neurology, Ruijin Hospital, School of Medicine, Shanghai Jiao Tong University, Shanghai 200025, China; 2 Department of Outpatient, Ruijin Hospital, School of Medicine, Shanghai Jiao Tong University, Shanghai 200025, China; 3 CAS Center for Excellence in Brain Science and Intelligence Technology, Ruijin Hospital, School of Medicine, Shanghai Jiao Tong University, Shanghai 200025, China

**Keywords:** Parkinson’s disease, intermittent theta burst stimulation (iTBS), neuroprotection, delayed-start, paired-pulse TMS, multi-modal

## Abstract

Background: As a typical high-disability neurodegenerative disease, Parkinson’s disease (PD) progresses variably, and patients who are clinically insensitive to dopaminergic therapy and whose symptoms fail to improve are commonly observed. As a result, achieving early neuron protection is critical. Methods/Design: The NET-PD study is a 2-year prospective single-center, double-blind, multi-arm, delayed-start, sham-controlled clinical trial assessing the long-term neuroprotective effect of intermittent theta burst stimulation (iTBS) in PD patients. Patients diagnosed with PD, aged 50–80, Hoehn–Yahr stage ≤4, and who maintain medication stability during the study will be enrolled. Clinical assessment and multi-modal markers are used to clarify the clinical improvement and dynamic neuronal changes in PD patients. With a standard deviation of 2, a test level of 0.05, a dropout rate of 10%, and a degree of certainty of 0.9, 60 PD patients are required for this study. Results: The NET-PD project was funded in March 2022, data collection began in July 2022, and is currently in the recruitment phase with two PD patients already enrolled. Data collection is expected to be completed in June 2024. The results are expected for publication in December 2024. Discussion: Previous research has demonstrated a rudimentary method for assessing and delaying PD progression in clinical medication trials. The NET-PD study adopts a rigorous methodology and specific disease-modifying designs to demonstrate the neuroprotective effect of iTBS on PD and investigate the potential mechanism of iTBS in regulating brain and motor functions. We hope to provide supposition for the subsequent exploration of diverse neuroprotection methods.

## 1. Introduction

Parkinson’s disease (PD) is a disabling neurodegenerative disease with core clinical features divided into motor symptoms (MS), which are characterized by bradykinesia, resting tremor, myotonia, and postural balance disorders, and non-motor symptoms (NMS), which are characterized by olfactory disturbances, sleep disturbances, autonomic dysfunction, and psychiatric disturbances [1]. The prevalence of PD increases substantially with age, with slightly more men than women. Worldwide statistics show a prevalence of 1–2% in people over 65 and 3–5% in people over 85 [2,3]. PD is currently treated with a combination of pharmacological therapy, surgery, exercise therapy, neuromodulation, psychological support, and caregiving. Pharmacotherapy, represented by carbidopa-levodopa, remains the primary treatment of choice [4]. Despite dopaminergic therapy being the first strategy for PD, patients who are clinically insensitive to medication and whose symptoms fail to improve are commonly seen. Surgical methods such as deep brain stimulation can improve motor symptoms in moderate-to-advanced PD. As a typical high-disability geriatric disease, PD progresses variably, and neither drugs nor surgery can effectively delay or stop the progression of the disease. Therefore, it is of great significance to strive for neuron protection early. Despite a number of large studies, no disease-modifying pharmacologic treatments have been identified at present [5].

With the discovery of structural and functional alterations in specific neural circuits associated with the development of PD, many researchers attempt to use neuromodulation targeting specific brain regions for the treatment of PD and have made some progress. High-frequency repetitive transcranial magnetic stimulation (HF-rTMS, ≥5 Hz), which induces increased local metabolism and enhances cortical activity, is considered as the most promising paradigm for treating motor function in PD [6,7,8,9,10]. As it is a regenerative stimulation pattern with a shorter stimulation duration and more intense stimulation sequence, intermittent theta burst stimulation (iTBS) still induces synaptic plasticity faster when paired with lower stimulation intensity [11]. A 6-hydroxydopamine (6-OHDA)-induced PD rat model revealed that the loss of nigrostriatal dopaminergic neurons was positively correlated with an iTBS-induced reduction in PD motor plasticity, and iTBS could induce the recovery of loss of dopaminergic neurons in substantia nigra [12]. iTBS in the primary motor cortex (M1) significantly improved limbic dyskinesia, reversed the reduction of dendritic spines, enhanced striatal excitability, modulated striatal plasticity, and induced long-time depression (LTD) in the striatum that are sensitive to low dopamine levels [13,14]. Additionally, within the PD rat brain, HF-rTMS synergistically neuronal repair soluble factors caused a favorable microenvironment for the survival of DA neurons with improved motor function [15]. Moreover, long-term iTBS seems to have a synergistic effect with dopaminergic neurotransmission or neuromodulation; specifically, iTBS on the dorsolateral prefrontal cortex increased the ipsilateral theta oscillatory power, which may contribute to the recovery of executive function and working memory [16].

Neuroprotective or disease-modifying therapy can slow or even stop disease progression and prevent the onset of eventual functional disability [17], which is critical for PD patients. Neuroprotective therapy faces a few challenges, the most important of which is the limitation of experimental design. The traditional washout trial design suffers from the disadvantages involved in uncertain washout period and high patient dropout rate [18]. Delayed-start trial designs can largely solve this nuisance. This kind of design eliminates the symptomatic effect brought by treatment itself, enabling more precise monitoring of the “protective effect” results [19]. Moreover, delayed-start design does not need a washout period, which allows more patients to be treated and is more ethical; thus, patients may be more cooperative [20]. Several large cohort studies devoted to neuroprotection and disease modification have initially confirmed the reliability of the delayed-start trial design, and the current results found little disease-modifying effects for most drugs [21,22,23].

To validate the therapeutic effectiveness of iTBS, large randomized controlled studies with long-term follow-up are still needed to validate the results of current studies. Additionally, TMS has not been explored in any study for its neuroprotective effects in PD patients, nor was there definitive evidence of PD neuroprotection. Based on the research background and previous foundation above, NET-PD proposes to adopt a delayed-start randomized double-blind pseudo-stimulation controlled study design to perform multi-sequential M1-iTBS under individual MRI-navigation to explore the ameliorative and neuroprotective effect of long-term treatment with iTBS on PD patients, and to explore the potential mechanism of its therapeutic action by assessing the relationship between dynamic changes in multi-modal indexes and clinical symptoms. The investigators hope to provide a possible way to protect or even reverse neuronal loss in PD and delay the progression of motor disorders in PD.

## 2. Methods/Design

### 2.1. Study Aims

The primary objective of NET-PD is to evaluate the neuroprotective effect of iTBS in PD patients. Secondly, clinical assessment and multi-modal markers are used to clarify the clinical improvement of motor symptoms and dynamic neuronal changes due to iTBS in PD patients, and to provide long-term follow-up evidence for the neuroprotective effect of iTBS. Meanwhile, by analyzing the dynamic changes before and after the treatment, we can explore the potential mechanisms of iTBS regulating brain and motor functions.

### 2.2. Study Design

**Figure 1:**
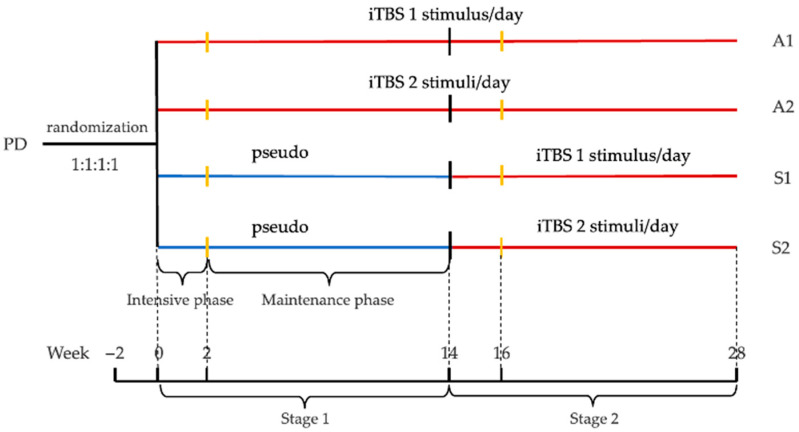
Schematic representation of study outline and visits. Stage 1 = pseudo-controlled stage, Stage 2 = active treatment stage; Phase 1 = intensive phase, Phase 2 = maintenance phase; A1 = early-start single iTBS group, A2 = early-start double iTBS group, S1 = delayed-start single iTBS group, S2 = delayed-start double iTBS group.

**Figure 2:**
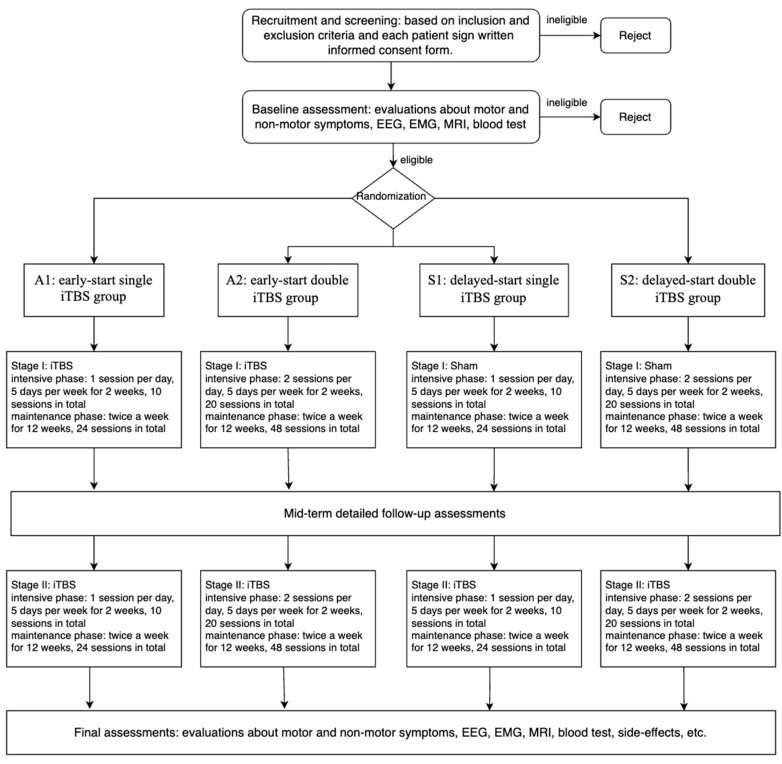
The NET-PD study flowchart.

NET-PD is a 2-year prospective single-center, double-blind, multi-arm, delayed-start, sham-controlled trial. The study is conducted in two stages—the pseudo-controlled and active-treatment stage—with each stage consisting of a 2-week intensive phase and a 12-week maintenance phase, for a total of 14 weeks. The intensive period involves daily or twice-daily iTBS (or pseudo-stimuli) per week, for a total of 10 or 20 sessions, and the maintenance period consists of 12 weeks of iTBS (or pseudo-stimuli) once or twice a day, 2 days a week, for a total of 24 sessions in the single-stimulation group or 48 in the double group. In stage 1 (pseudo-controlled stage), patients are randomly assigned to one of four intervention groups in a 1:1:1:1 ratio: early-start single iTBS group (A1), early-start double iTBS group (A2), delayed-start single iTBS group (S1), and delayed-start double iTBS group (S2). The early-start groups conduct active iTBS throughout, whereas the delayed-start groups use pseudo-stimuli in the first stage and start using iTBS in the second stage. The only difference between the single- and double-stimulation group is the number of sessions per day: once daily for the single group and twice daily for the double group. In stage 2 (active treatment stage), participants in A1 and A2 continue to receive the same iTBS protocol as the first stage, while those in S1 and S2 start to receive active iTBS. All patients are required to have a detailed follow-up clinical assessment and electrophysiology record at baseline, 2 weeks, 14 weeks, 16 weeks, and 28 weeks. MRI and peripheral blood are collected at baseline, 14 weeks, and 28 weeks (Figure 1, Table 1). Each patient will spend 7 months in this study and the total duration of the research will be approximately 2 years, from July 2022 (first in) to December 2024 (last out) (Figure 2).

### 2.3. Inclusion Criteria

Patients entering this study are required to meet all of the following criteria:

1. PD diagnosed according to the revised clinical diagnostic criteria of the Movement Disorder Society (MDS) International (2015 version);
2. Aged 50–80, age of diagnostic ≥50, male or female;
3. Hoehn–Yahr stage ≤4;
4. With or without levodopa, maintaining medication stability during the study period;
5. Good compliance with the long-term intervention and follow-up.

Written informed consent should be signed by each patient; this study has been approved by Ruijin Hospital’s ethical committees.

### 2.4. Exclusion Criteria

Subjects meeting any of the following criteria will be excluded from this study:

1. Presence of any of the features that rules out PD (e.g., unequivocal cerebellar abnormalities, downward vertical supranuclear gaze palsy, selective slowing of downward vertical saccades etc.);
2. Patients with severe mental illness or neurological disorders (e.g., epilepsy, cerebrovascular accidents, etc.) or a history of traumatic brain injury or brain surgery;
3. Patients with significant cognitive impairment (MMSE <24) or inability to complete questionnaires independently;
4. Previously treated with TMS, deep brain stimulation (DBS) or spinal cord stimulation (SCS);
5. Have any physical illness that can precipitate epilepsy or intracranial hypertension, including cardiovascular and respiratory disease;
6. Have human implantable materials such as intracranial stents, pacemakers, coronary stents, cochlear implants, etc.;
7. Are currently taking other investigational drugs or participating in other clinical trials;
8. Any other condition that the investigator deems unsuitable for this study.

### 2.5. Withdrawal Criteria

Subjects may withdraw at any time if anything occurs that may jeopardize their interests. Subjects who discontinue the study will no longer be covered by continued data collection. The reason for termination or withdrawal should be documented in the case report form. Patients should withdraw from the trial immediately if they meet the following criteria:

1. Poor compliance to the protocol;
2. Occurrence of an intolerable adverse event;
3. Patients decline further treatment or follow-up;
4. Loss of visits due to unavoidable situations, such as death;
5. The investigators terminate the subject’s continued participation after review.

### 2.6. Endpoint Criteria

Participants are considered to have completed the experiment when they finish all treatments and assessments or withdrew from the experiment for any reason midway through.

### 2.7. Recruitment and Screening

In total, 60 PD patients will be enrolled from the Parkinson’s Disease and Movement Disorders Clinic of Ruijin Hospital in Shanghai. Subjects who have been clinically diagnosed with PD by two specialists independently are recruited and screened based on inclusion and exclusion criteria. Cognitive function assessed by Mini-Mental State Examination (MMSE) and a history of neurological disorders and treatment are the focus of screening. Additionally, a blood test and structural MRI should be performed to exclude patients with other unsuited conditions for the study.

### 2.8. Randomization and Blinding

To reduce measurement bias during subjective assessment, we adopt a randomized grouping and double-blind design. Participants are assigned into four arms in a 1:1:1:1 design according to a predetermined generated random number table generated by computer algorithm before the start of the entire experiment. The clinical assessments are scored by specialists not involved in the intervention and grouping, based on the coded and disordered video to ensure the objectivity and authenticity. All the patients are blind to treatment. The pseudo-stimulation coil looks exactly the same as the real iTBS coil and emits the same sound and vibration sensation during the stimulation process, but there is no actual stimulation effect. Coil replacement will be completed before the patient enters the treatment room, which ensures that subjects are unaware of the grouping. Specifically, iTBS operators are unblinded because they need to exchange coils to conduct both active and sham stimuli. Blinding will be evaluated by asking participants to guess group assignment at the end of the whole experiment.

### 2.9. iTBS Intervention and Follow-Up

During iTBS, three consecutive 50 Hz pulses are embedded in 5 Hz pulses (200 ms interval). The pattern is then repeated every 10 s in 2 s stimuli followed by 8 s rest sequence, for a total of 600 pulses in each session. In this study, the NS5000 transcranial magnetic stimulator and accompanying devices manufactured by Wuhan Yiruide New Technology Ltd. are used for treatment and measurements. Like the placebo control group in drug experiments, the pseudo-stimuli group is the control group to the active iTBS group. Active and pseudo-stimuli are delivered by matched coils, and a special pseudo-stimuli coil with the same appearance and sound as the active coil will be used. Patients can hear the specially designed sound and feel the vibration during stimulation, but there is no actual stimulation effect. This ensures that subjects are unaware of the grouping. Based on previous results, iTBS intervention was well tolerated with only few subjects reporting discomfort or pain, and no pathological increases in cortical excitability or seizure activity recorded on EEG/EMG monitoring [24,25,26].

Throughout the intervention, patients are first instructed to sit stably and relax in a comfortable position. Before the first intervention, each patient’s resting motor threshold (RMT) should be measured, which is defined as the minimum stimulus intensity necessary to elicit an overt motor response in the abductor pollicis brevis for ≥50% of applied stimuli [27]. We deliver iTBS to the bilateral primary motor cortex (M1) at 100% RMT. Stimulation targets for bilateral M1 are localized according to individual MRI brain structure images under the visor2 ^TM^ neuro-navigated system (ANT Neuro Ltd., Hengrlo, The Netherlands). Subjects in each arm undergo two intervention circles, each composed in an intensive phase for 2 weeks, followed by a maintenance phase of twice-weekly sessions for 12 weeks. Each patient should be stimulated at the same time of day to minimize the interference of disturbing factors. The stimulation interval between two stimuli in double-stimulation groups is 60 min [28], which could maximize the long-term potentiation (LTP) of synapses.

### 2.10. Paired Pulse TMS (ppTMS) Strategy

Paired-pulse transcranial magnetic stimulation (ppTMS) is a non-invasive method utilized to measure GABAergic activity within M1 to probe inhibitory and excitatory networks, which is a common TMS-combined test that allows for quick and easy measurements before and after treatment [29,30]. Before having iTBS, we use a paired-pulse TMS (ppTMS) strategy to investigate intracortical inhibitory and excitatory functional connections within M1, including short-interval intracortical inhibition (SICI), long-interval intra-cortical inhibition (LICI), and intracortical facilitation (ICF) [31]. To conduct ppTMS, a hand-held figure-of-eight coil and NS5000 transcranial magnetic stimulator are used (Wuhan Yiruide New Technology Ltd., Wuhan, China). After pre-test resting EEG, RMT of each subject will be tested, which is the minimum stimulus intensity triggered by TMS pulse in right M1 evoking at least five responses (above 50 μV) out of 10 stimuli. Motor-evoked potential (MEP) can be elicited by ipsilateral M1 stimulus and obtained using surface EMG recording electrodes settled on contralateral hand. SICI and ICF are assessed through ppTMS with a subthreshold conditioning stimulus (CS) at 80% of RMT followed by a suprathreshold testing stimulus (TS) at 120% of RMT according to standard protocols [32,33]. The interstimulus interval (ISI) between the CS and TS determines whether the cortex produces inhibitory or excitatory activity [34]. In most cases, SICI attenuates the MEP response when a subthreshold CS precedes a suprathreshold TS by 1–6 ms, while ICF facilitates the MEP response when a subthreshold CS precedes a suprathreshold TS by 8–30 ms [32,35]; thus, our experiment adopts ISI = 5 ms and ISI = 20 ms for SICI and ICF measurement, respectively. Additionally, LICI is typically tested by applying suprathreshold CS at 120% of RMT followed by TS at 120% of RMT at ISI = 200 ms, resulting in a reduced MEP amplitude [31]. All the measurements will be performed first in the more affected hemisphere and then in the less affected hemisphere.

### 2.11. Neuroimaging

Structural and functional images are acquired with 3T Siemens scanners with a 12-channel head coil at baseline, 14-week follow-up, and endpoint. T1-weighted images are obtained using a 3D magnetization prepared rapid acquisition gradient-echo (MPRAGE) sequence, setting slices = 192, field of view = 250 mm, thickness = 1 mm, flip angle = 9°, voxel size = 0.5 × 0.5 × 1 mm^3^, echo time = 2.44 ms, repetition time = 1900 ms, and inversion time = 900 ms for location purposes. Regarding resting functional MRI, we use the blood-oxygen-level-dependent (BOLD) signal to identify neural network changes carried out by iTBS related with motor and non-motor performance, with slices = 36, field of view = 192 mm, thickness = 3 mm, flip angle = 90°, voxel size = 3.0 × 3.0 × 3.0 mm^3^, echo time = 22 ms, repetition time = 2000 ms, and inversion time = 900 ms. Neuroimaging will be scanned at baseline, at the end of stage 1, and at the end of stage 2.

### 2.12. Electroencephalogram (EEG) and Electromyographic (EMG)

Resting EEG signals are received using EBneuro Beplus pro EEG 64-channel recording system (EBneuro, Florence, Italy) in a separate, quiet room. Sixty-four-channel EEG signals are sampled at a frequency of 1024 Hz, and a 50 Hz notch filter is applied to reduce the input noise. The individuals are instructed to seat comfortably in a stable chair with both hands relaxed during the recordings. The skin is cleaned at the electrode contacts with alcohol before attaching the 64-channel-customized EEG cap to reduce skin impedance. The distribution of the electrode gel is applied and adjusted with a flat syringe and cotton swab until all electrodes reach the desired impedance (<10 kΩ). Two sessions of resting EEG will be performed before and after the iTBS stimulus, which consist of 5 min of open eyes and 5 min of closed eyes. With 9 mm-diameter surface electrode patches, EMG traces will be acquired bilaterally from the abductor pollicis brevis (APB) muscles. EMG acquisition will be performed through the EMG module that affiliates to the iTBS stimulator. EEG and EMG measurements will be collected at baseline, week 2, midterm (week 14), week 16, and endpoint (week 28).

### 2.13. Blood Test

Peripheral blood will be collected at baseline, midterm (week 14), and endpoint (week 28). At the screening stage, 10 mL of peripheral blood will be drawn for routine laboratory tests, including blood cell sorting count, platelet count, hemoglobin level, liver and kidney function, blood creatinine, fasting glucose, and coagulation function tests. Peripheral blood will be drawn through a vein and collected into EDTA tubes. Within 30 min of collection, the supernatant is separated by centrifugation at 3000× *g* for 10 min at 4 °C to obtain plasma. The plasma is then packed into Eppendorf (EP) tubes of 0.5 mL each and stored at −80 °C for subsequent batch monitoring. Exosome alpha-synuclein (α-syn) will be measured using plasma. As a first step, antibody-coated superparamagnetic microbeads are used to isolate exosomes from human plasma [36]. Plasma samples are mixed with buffer A and buffer B and then diluted with phosphate-buffered saline (PBS), and the mixture is then incubated with dynabeads on a rotator at 4 °C for 1 h. Incubation of the mixture is continued with PBS on a rotator at 4 °C for 15 min and the supernatant containing plasma exosomes is collected. Plasma exosomes are characterized according to size and shape using transmission electron microscopy (TEM). The size of the sample is determined directly by nanoparticle tracking analysis (NTA) using a NanoSight LM10 microscope (Malvern Panalytical Ltd., Enigma Business Park, Grovewood Road, Malvern, UK). The purity of positive and negative exosome markers is verified using Western blotting. Finally, the U-PLEX Human-Synuclein Kit (Meso Scale Discovery Co., Rockville, MD, USA) on the Quick Plex SQ120 platform is used to detect α-syn levels [37,38].

### 2.14. Baseline Assessments and Follow-Up Evaluations

After screening, all the eligible patients will undergo a comprehensive detailed assessment, including basic demographic, motor and non-motor symptoms, α-syn in the peripheral blood, and resting functional MRI, EEG, and EMG. All the physicians involved in assessment receive consistency training prior to the start of the trial to ensure the uniformity of the standard. Basic demographic information including gender, age, education, concomitant diseases, family history, medication usage, etc. is taken. All motor symptom assessments will be video-numbered and scored by two independent physicians. Various aspects of the patients’ clinical symptoms are measured using Unified Parkinson Disease Rating Scale (UPDRS), Berg Balance Scale (BBS), Hamilton Depression Scale (HAMD), Hamilton Anxiety Scale (HAMA), MMSE, Montreal Cognitive Assessment (MoCA), Pittsburgh sleep quality index (PSQI), 39-item PD Questionnaire (PDQ-39), 16-item odor identification test from Sniffin’ Sticks (SS-16), Scale for Outcomes in Parkinson’s disease for Autonomic Symptoms (SCOPA-AUT), and Wexner constipation scale. At the same time, we draw the patient’s peripheral blood for cytology, biochemistry, and coagulation tests and to detect α-syn in exosomes. After two intensive phases (week 2 and week 16), patients will receive a brief follow-up evaluation including the UPDRS, EMG acquisition and side effects. At midterm (week 14) and the endpoint of the experiment (week 28), the patients must complete the same detailed set of evaluations as the baseline.

### 2.15. Outcome Measures

According to the results of previous large delayed-start design disease-modifying drug studies, the neuroprotective effect can be commonly reflected by the difference in the changes in the UPDRS score before and after treatment between groups [22,39]. In an NET-PD study, the primary outcome is the differences in changes in UPDRS scores in the four groups before and after the iTBS intervention. As a secondary outcome, we will evaluate the effects of iTBS treatment on clinical symptoms, motor symptoms such as gaiting and postural balance, and non-motor symptoms such as mood, cognition, olfaction, and sleeping condition in PD patients. As a neuro-modulation intervention study, EEG, EMG and MRI images can also be visualized to indicate the functional activity status of the brain. The safety of the treatment will be assessed using a self-administered side effect scale. Additionally, self-administered side effects scales will be used to assess iTBS safety.

### 2.16. Study Hypothesis

Firstly, UPDRS scores are expected to be significantly lower in group A1 and A2 than in group S1 and S2 after stage 1, and the scores show a trend from low to high in groups A2, A1, S2, and S1 after stage 2. Next, the change in the slope of the weekly UPDRS scores will be compared among the 4 groups. We expect a slower rate of deterioration (i.e., increase in UPDRS scores) with the iTBS intervention than with the pseudo-stimulation group. The mean total UPDRS score deteriorates less from baseline to week 28 in the early-start groups than in the delayed-start groups. For the changes in slope of UPDRS scores between weeks 14 and 28, the early-start groups are expected to respond better than the delayed-start groups.

### 2.17. Sample Size and Statistical Analysis

With reference to data from previous studies published in relevant journals, a total of 60 PD patients are proposed to be enrolled in this single center study. The sample size of this study was calculated based on the improvement in the UPDRS scale after 28 weeks of treatment and differences between groups. Calculations are performed using PASS (Power Analysis and Sample Size) 2008 software (UT, OH, USA), assuming a four-point reduction in the UPDRS at the end of treatment in A1, a five-point reduction in A2, a two-point reduction in S1, and a four-point reduction in S2, with a standard deviation of 2, a test level of 0.05, and a degree of certainty of 0.9 yielded for 13 subjects per group. Considering that the patient dropout rate may be about 10% [24,40], the final plan is to enroll n = 15 cases in each group for n = 60 cases in total. The minimum sample size required to meet the hypothesis of this clinical study is 60, and actual samples of 60 cases or more are in accordance with the clinical study design principles and statistical requirements.

Statistical analysis applies SPSS 20.0 for processing; MRI, EEG, and EMG data are processed on MATLAB platform (Math Works Inc., Natick, MA, USA). All statistics are performed using two-sided tests, and α = 0.05 is the cut-off point used to decide whether the hypothesis is statistically significant. Between-group comparisons of quantitative data will be performed using analysis of variance (ANOVA) or Wilcoxon rank sum test depending on the distribution of the data. A paired *t*-test or Wilcoxon signed-rank test will be used for comparison before and after treatment. Categorical data are tested by chi-square test or exact probability method (if the chi-square test is not applicable).

To compare the changes in UPDRS scores before and after the iTBS intervention at each time point, mainly comparing the differences in their means, two-way repeated-measures ANOVA was used to compare the main effects and the interaction effects between the two for different groups and follow-up points (baseline, week 14, week 28), and post hoc analysis was performed using the Bonferroni test to compare the differences between each follow-up point and baseline scores.

## 3. Results

The NET-PD project was funded in March 2022; data collection began in July 2022 and is currently in the recruitment phase, with two PD patients already enrolled. Data collection is expected to be completed in June 2024, and data analysis in October 2024.The results are expected for publication in December 2024.

## 4. Discussion

The cumulative frustration of TEMPO [23] and ADAGIO [41] investigations exemplifies the rough way to assess and postpone PD progression in clinical drug trials. Another challenge in developing clinical trials for neuroprotection is that in vivo brain neuronal counts are not available; therefore, no methods exist to monitor neuroprotection in the clinic. A TMS intervention and a delayed-start design may be able to address some of these problems. Several clinical trials have demonstrated the therapeutic benefits of TMS and TBS.As a novel form of excitatory TMS protocol, iTBS is less time-consuming and more effective than standard rTMS [42]. Another advantage is that TMS can be paired with EEG and EMG at the same time to monitor neuronal excitability as well as cortical function for PD patients. As well as better interpreting disease-modifying effects, delayed-start designs are reliable experimental designs for assessing neuroprotection. Once a month, the investigators, PD experts, and ethics committee will meet in person or online to discuss the trial’s progress and patient’s safety. The NET-PD trial is led by the Department of Neurology, Ruijin Hospital (PI: Jun Liu), and is under the supervision of the Clinical Research Center, Shanghai Jiao Tong University School of Medicine.

Through this delayed-start, randomized, double-blind, sham-controlled trial, we hope to quantify the neuroprotective effect of iTBS in PD patients.

## Figures and Tables

**Table 1:** Outline and timelines of the trial.

	Screening	Baseline	Visit 1	Visit 2	Visit 3	Visit 4
	−2 Weeks	0	2 Weeks	14 Weeks	16 Weeks	28 Weeks
Written informed consent	×					
Inclusion/exclusion criteria	×					
Randomization		×				
Basic demographic		×				
Hoehn–Yahr stage		×	×	×	×	×
UPDRS		×	×	×	×	×
BBS		×		×		×
HAMD		×		×		×
HAMA		×		×		×
MMSE	×			×		×
MoCA		×		×		×
PDQ-39		×		×		×
PSQI		×		×		×
SS-16		×		×		×
SCOPA-AUT		×		×		×
Wexner		×		×		×
MRI	×	×		×		×
EEG		×		×		×
EMG		×	×	×	×	×
Blood test	×	×		×		×
Compliancy		×		×		×
Medication usage	×	×	×	×	×	×
Side effects			×	×	×	×

×, conducting evaluations.
